# Effects of Cold Decompression on Hemodynamic Function and Decompression Sickness Risk in a Dry Diving Rat Model

**DOI:** 10.3389/fphys.2021.763975

**Published:** 2021-11-03

**Authors:** Svein E. Gaustad, Timofei V. Kondratiev, Ingrid Eftedal, Torkjel Tveita

**Affiliations:** ^1^Møreforsking AS, Volda, Norway; ^2^Department of Circulation and Medical Imaging, Norwegian University of Science and Technology, Trondheim, Norway; ^3^Anesthesia and Critical Care Research Group, Department of Clinical Medicine, UiT, The Arctic University of Norway, Tromsø, Norway; ^4^Faculty of Nursing and Health Sciences, Nord University, Bodø, Norway; ^5^Division of Surgical Medicine and Intensive Care, University Hospital of North Norway, Tromsø, Norway

**Keywords:** diving, hemodynamic function, temperature, cold diving, left ventricle, venous gas bubbles

## Abstract

**Background:** Diving in cold water is thought to increase the risk of decompression sickness (DCS), especially if the diver is cold during decompression. In this study, we investigated hemodynamic function and DCS risk in an animal model, where cold decompression was followed by rewarming at the surface.

**Methods:** Nine female Sprague Dawley rats had pressure-volume catheters inserted into their left heart ventricle and femoral artery before they were exposed to dry air dives in which their core temperature was normothermic during the bottom phase, cold (35°C) during decompression, and normothermic after the dive. Data from an earlier study were used as controls. The rats were compressed in air to 600kPa, maintained at pressure for 45min, and decompressed at 50kPa/min. Hemodynamic data were recorded before, during, and 60min after decompression. Venous gas bubbles were recorded in the right heart ventricle and pulmonary artery for 60min after the dive.

**Results and Conclusion:** During decompression, cardiac output (CO), and stroke volume (SV) decreased equally in cold rats and controls. CO and SV were temporarily re-established at the surface, before falling again in the cold rats. There was no difference in post-dive venous bubble grades. However, as the post-dive fall in CO and SV could be a sign of gas emboli obstructing the pulmonary circulation, we cannot conclude whether the DCS risk was increased. More sensitive bubble detection methods are needed to elucidate this point.

## Introduction

DCS risk after diving is thought to be temperature dependent, although causal relationships are undetermined (Dunford and Hayward, 1981; Mekjavić and Kakitsuba, 1989; Leffler, 2001; Fahlman and Kayar, 2006). Empirical and experimental data indicate that cold water diving is associated with more DCS, especially if the diver is cold during decompression (Toner and Ball, 2004). United States Navy procedures require longer decompression times when divers are “exceptionally cold” (Commander, 1999).

DCS is a multifaceted disease triggered by inert gas bubbles released from supersaturated tissues during decompression. The uptake and removal of inert gas is determined primarily by tissue perfusion (Ohta et al., 1978), which is regulated by temperature sensitive mechanisms (Barcroft and Edholm, 1943; Caldwell et al., 2016). It follows that a diver who is cold during the bottom phase and warm during decompression would desaturate more efficiently and therefore may have lower DCS risk compared to one who is warm at the bottom, cold during decompression, and rewarmed at the surface (Dunford and Hayward, 1981; Mack and Lin, 1986; Mekjavić and Kakitsuba, 1989; Gerth et al., 2007). In addition to temperature, tissue perfusion depends on the solubility and diffusion capacity of the breathing gas (Lango et al., 1996), and the picture is further complicated by other factors, which contribute to DCS risk, including immersion, BMI/fat mass, age, gender, and work load/activity levels before, during, and after the dive (Dujic et al., 2006, 2008; Cialoni et al., 2017). In this complex picture, there is a need for controlled experiments that focus on thermal effects.

In this comparative study, we examined whether a core temperature reduction to 35°C during decompression followed by rewarming to 37°C at the surface would increase the risk of DCS. We employed a previously established diving rat model designed to measure hemodynamic function (Gaustad et al., 2020), to which we added core temperature control and used post-decompression venous gas bubble formation as a proxy measure for DCS.

## Materials and Methods

### Ethics

The study protocol was approved in advance by the Norwegian Council for Animal Research (approval ID 2111). All procedures were consistent with the European Convention for the Protection of Vertebrate Animals used for Experimental and Other Scientific Purposes. In partial fulfillment of the requirement to minimize the number of animals, the control rats referred to in this experiment are the same individuals as previously described (Gaustad et al., 2020).

### Animals

Female Sprague Dawley rats (259.0±5.4g) were obtained from Charles River Laboratories (Charles River Laboratories Inc., Sulzfeld, Germany). To limit stress to the animals prior to the dives, they were kept at an approved animal facility in which they had *ad libitum* access to a standard rodent chow and water and were handled by a dedicated technician. As rats are nocturnal, the light in the facility was controlled at a 12-h dark-12h cycle with the dark cycle coinciding with human daytime. The experimental dives were performed during the rat’s wake (dark) cycle.

### Pre-dive Procedures

Prior to the experiment the rats were anesthetized by sodium pentobarbital. A 13 Gauge metal tube was inserted into the trachea to establish a patent airway. For hemodynamic monitoring, two microtip pressure-volume (P-V) catheters (SPR-838, 2.0 F, Millar Instruments; Houston, TX, United States) were used, one was inserted into the left ventricle (LV) to obtain LV volumes and LV pressure and another one was placed into the femoral artery to obtain MAP as previously described (Gaustad et al., 2020). The rats rested for 60min to regain hemodynamic stability and were placed in the supine position and kept under anesthesia while breathing spontaneously until the end of the experiment.

### Conductance Measurement Calibration for Hypothermia

The principle for adjustment of conductance catheter readings for different temperatures was previously described in detail by Han et al. (2008). In short, for assessment of the left ventricular volume, the cuvette calibration was used to adjust for temperature-dependent changes in blood viscosity. Plexiglas calibration cuvettes (910–1048, Millar Instruments, Houston, TX, United States) with wells of known volumes from 28 to 346μl were filled with heparinized blood from the rat and immersed in a temperature-controlled water bath to adjust blood temperature to 37, 36, and 35°C during catheter calibration. The P-V catheter was sequentially placed into wells, and the conductance values were recorded for each well at each temperature mentioned above. Linear regression between conductance values and corresponding wells’ volumes was plotted for each temperature. Slopes and y-intercepts obtained at the above temperatures were applied to the analysis software (PVAN 3.6, Millar Instruments, Houston, TX, United States) to convert conductance units to true volumes in μl. Ideally, measurements should be corrected for parallel conductance induced by the alternating current passing from the catheter through the blood and into the surrounding LV wall or inter-ventricular septum. Parallel conductance is usually measured by a saline bolus injection at the end of experiment (Georgakopoulos and Kass, 2000). However, we did not consider this method suitable to our experimental protocol, which required the measurements of left ventricular volume at different temperatures. Considering temperature-dependent changes in viscosity of blood, a bolus injection performed at the end of experiment (37°C) would not represent the true parallel conductance at lower core temperature, and injection of hypertonic saline (30% NaCl) in animals positioned inside the chamber was not possible. In addition, repeated injections might harm the animal. Therefore, parallel conductance correction was not included in our volume measurements.

### Core Temperature Control

An electric heat pad was used to control the rats’ core temperature throughout the experiment. For temperature monitoring, we used a digital thermometer (Thermoalert, Columbus Instruments, OH, United States) connected to a thermocouple wire with its tip positioned in the lower 1/3 of the rat’s esophagus. During the simulated diving exposure, the thermometer was placed inside the pressure chamber and observed through a porthole.

### Simulated Diving

**Figure 1:**
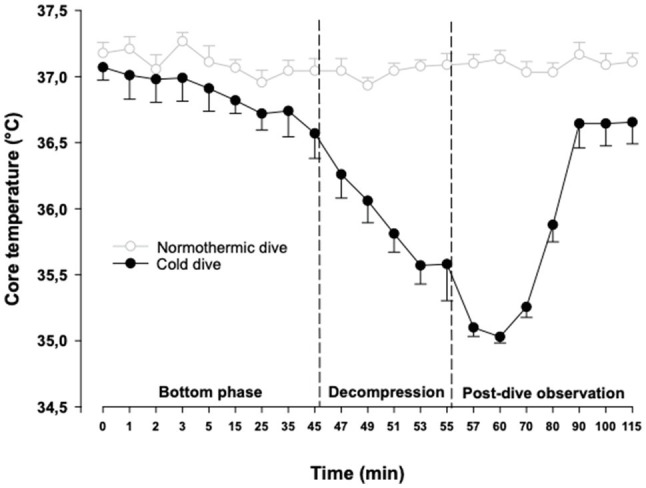
Body core temperature. The cold dive group was normothermic during the bottom phase, cooled to 35°C during the decompression and rewarmed to 37°C after the dive. Temperatures are mean±SEM. *N*=9 in both groups. Data for the normothermic control dive group are adapted from Gaustad et al. (2020).

The protocol for simulated diving has been described in detail elsewhere (Gaustad et al., 2020). In short, rats (*n*=9) were exposed one at a time to hyperbaric air in a pressure chamber (Sira Engineering, Trondheim, NO). Compression was done at 200kPa/min to a pressure of 600kPa, corresponding to a water depth of 50m. This pressure was maintained during a 45-min bottom phase before the animals were decompressed back to surface at 50kPa/min over 10min. The dive was followed by a 60-min post-dive observation. During the bottom phase, the rats were kept at a normothermic core temperature (37°C). They were cooled to 35.1±0.2°C during the decompression, before they were gently rewarmed to normothermia for the 60min post-dive observation (Figure 1).

### Hemodynamic Data Recording and Analysis

The following data were obtained during the experiment: mean arterial pressure (MAP), heart rate (HR), maximal LV systolic pressure, LV end-diastolic pressure, maximal slope of LV systolic pressure increment (LV dP/dtmax), stroke volume (SV), LV end-diastolic volume (LVEDV), LV end-systolic volume (LVESV), CO, and stroke work (SW). Preload recruitable stroke work (PRSW) was measured before and after the simulated diving. During the bottom phase of the dive, recordings were done at 1, 2, 3, and 5min, and further repeated every 10min. During the decompression, recordings were done every 2min, and during the post-dive observation, recordings were done after 2min, 5min, and then repeated every 10min until the end of the experiment after 60min. The data were recorded using ADInstruments LabChart DAQ software (AD Instruments, Hastings, United Kingdom) and analyzed in a cardiac P-V analysis program (PVAN 3.6, Millar Instruments, Houston, TX, United States).

### Post-decompression Venous Gas Bubbles

Venous gas bubbles were detected by insonating the rat’s pulmonary artery and aorta using a GE Vingmed Vivid-i ultrasonic scanner with a 10-MHz transducer to record as previously described (Wisloff and Brubakk, 2001). Bubbles were recorded at 10-min intervals up to 60min, at which time the animals were sacrificed. Ultrasound images were used to grade bubble loads by the Eftedal and Brubakk scale (Eftedal and Brubakk, 1997).

### Statistics

Hemodynamic data were assessed by one-way repeated measures ANOVA. In cases where the *F* value was greater than critical, Dunnett’s test was used to evaluate differences between baseline and later measurements. Mann–Whitney U test was used to evaluate differences in bubble grades. Effects were considered significant for *p*<0.05. Results are shown as mean±SEM.

## Results

### Hemodynamics

**Figure 2:**
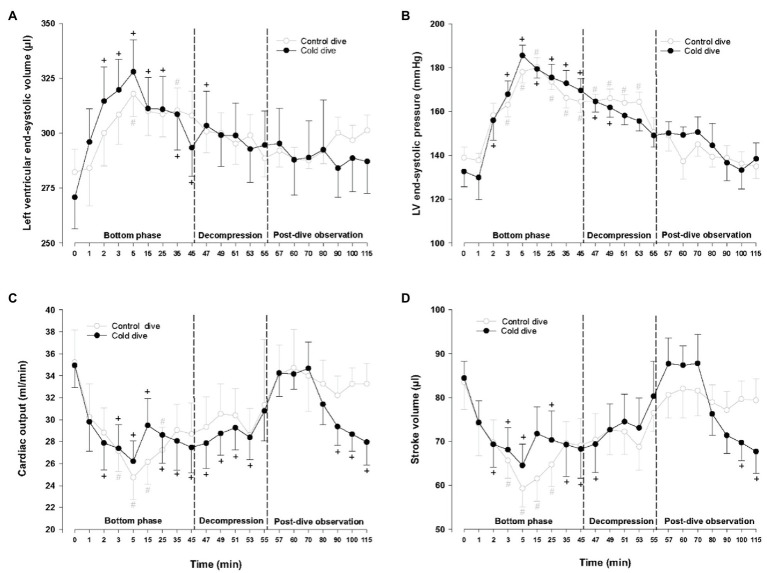
Hemodynamic variables. **(A)** Left ventricular end-systolic volume (LVESV), **(B)** left ventricular end-systolic pressure (LVESP), **(C)** cardiac output (CO), and **(D)** stroke volume (SV). Data are mean±SEM. *N*=9 in both groups. ^+^*p*<0.05 compared to baseline in the cold dive group. ^#^*p*<0.05 compared to baseline in the control dive group. Data for the control dive group are adapted from Gaustad et al. (2020).

**Figure 3:**
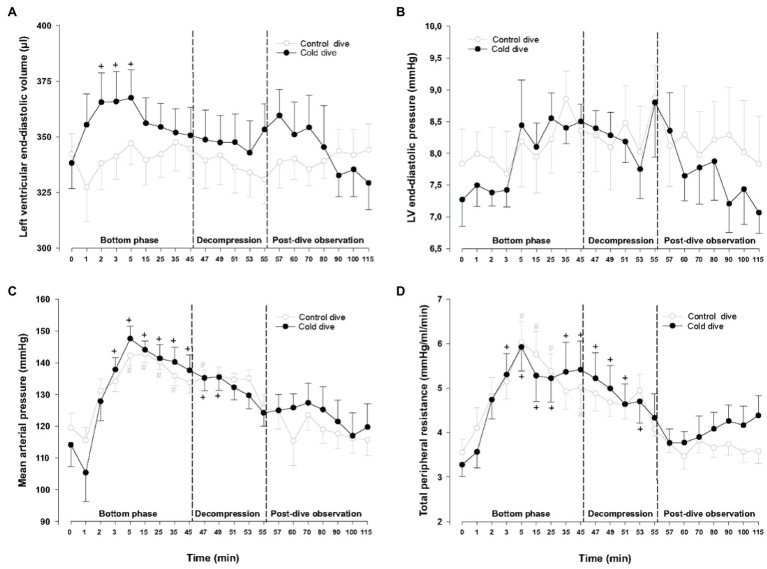
Hemodynamic variables (cont.). **(A)** Left ventricular end-diastolic volume (LVEDV), **(B)** left ventricular end-diastolic pressure (LVEDP), **(C)** mean arterial pressure (MAP), and **(D)** total peripheral resistance (TPR). Data are mean±SEM. *N*=9 in both groups. ^+^*p*<0.05 compared to baseline in the cold dive group. ^#^*p*<0.05 compared to baseline in the control dive group. Data for the control dive group are adapted from Gaustad et al. (2020).

**Figure 4:**
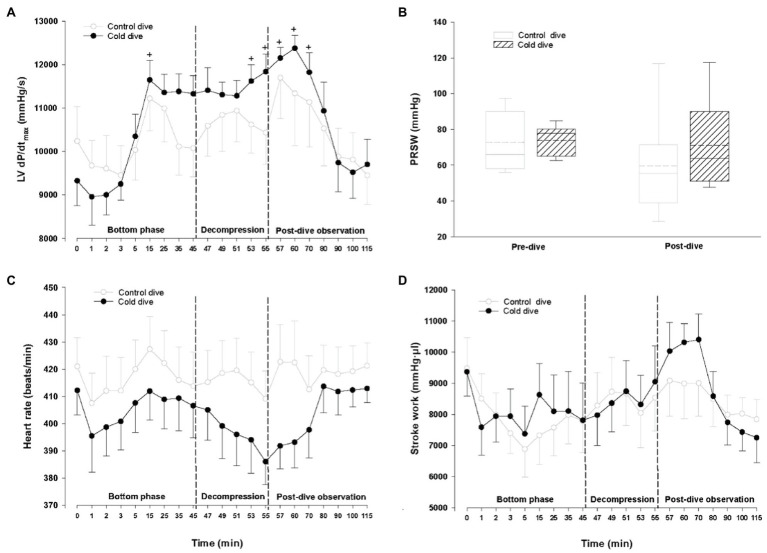
Hemodynamic variables (cont.). **(A)** Maximal slope of systolic pressure increment (LV dP/dt_max_), **(B)** preload recruitable stroke work (PRSW), **(C)** heart rate (HR), and **(D)** Stroke work (SW). Data presented as mean±SEM for **(A,C)**, and **(D)**. Data are presented as vertical boxes with median (solid line), mean (dashed line) and interquartile range with 10^th^ and 90^th^ percentile error bars for **(B)**. *N*=9 in both groups. ^+^*p*<0.05 compared to baseline in the cold dive group. ^#^*p*<0.05. Data for the control dive group are adapted from Gaustad et al. (2020).

During the bottom phase of the dive, LV end-systolic volume and pressure increased significantly after 2min, peaked after 5min (+21 and +40%, respectively), returned to pre-dive levels during decompression, and remained unchanged during the post-dive observation period (Figures 2A,B). Simultaneously, a significant decrease in CO and SV (−26 and −232%, respectively) took place, returned to control during decompression, but unlike all other cardiac variables a significant reduction of both CO and SV re-emerged during the final part of the 60min post-dive observation period after the animals had been rewarmed to normothermia (Figures 2C,D). Whereas LV end-diastolic volume was significantly elevated during the dive for the short period when LV-systolic pressure peaked, LV end-diastolic volume remained unchanged (Figures 3A,B). The changes in LV – functional variables took place in parallel with a significant increase in MAP and TPR, which returned to control during decompression (Figures 3C,D). LVdP/dt_max_, which was continuously monitored (Figure 4A), showed increase in LV-contractility during the last period of decompression and the early part of the post-dive observation; whereas PRSW, which was measured before the dive and at the end of the experiment, showed no change in contractility (Figure 4B). HR and SW were unaltered relative to control throughout the experiment (Figures 4C,D).

### Venous Gas Bubbles

There were no differences in venous bubble grades after decompression when the cold diving rats in this study were compared to normothermic diving rats from Gaustad et al. (2020) (Table 1).

**Table 1:** Minimal core temperature during decompression vs. maximal bubble grades observed post-dive in rats exposed to simulated diving.

Group	Minimal core temperature (°C)	Bubble grades
^*^Normothermic	37.2±0.3	0 (0–2)
Cold decompression	35.1±0.2	0 (0–3)

Minimal core temperatures are mean±SEM, bubble grades are median and range. *N*=9 in both groups.

* Data for the normothermic group are from Gaustad et al. (2020).

## Discussion

In this study, we used P-V catheterized and temperature-controlled rats to investigate hemodynamic function and DCS risk in response to a 2°C core temperature reduction during decompression from a simulated dive followed by rewarming at the surface. To control for specific effects of cooling, the hemodynamic data were controlled against our previously reported data on normothermic animals (Gaustad et al., 2020). Like in the normothermic controls, the rats in the present study experienced a physiologic reduction in CO and SV during the dive, caused by an abrupt increase in LV afterload induced by the hyperbaric environment. Ultrasound insonation with a 10MHz probe revealed no changes in bubble grades to indicate increased DCS risk. However, after the animals in the current study were rewarmed at the surface, they experienced a second fall in CO and SV that was not present in the controls.

Why did CO and SV fall after the animals were rewarmed? The post-dive reduction in SV cannot have been caused by reduced LV mechanical function. On the contrary, the absence of change in the indexes of cardiac contractility, dP/dt_max_ and PRSW, makes it unlikely that the reduction in SV and CO were caused by myocardial dysfunction. Neither is it likely that the outcome was triggered by the changes in core temperature: a significant reduction in CO after rewarming has been reported in animals after rewarming from profound hypothermia to 15°C (Kondratiev et al., 2006), but the modest 2°C temperature reduction in our study is above the threshold at which changes in hemodynamic function have been reported (Reuler, 1978; Danzl and Pozos, 1994).

One possible explanation is that the post-dive fall in CO and SV is the consequence of an abrupt increase in pulmonary resistance and subsequent reduced LV volume load. This is supported by a concurrent, albeit non-significant, trend toward the reduction of both LV end-diastolic pressure and volume (Figures 3B, 4B). Gas emboli can cause increased pulmonary resistance (Mélot and Naeije, 2011). Pulmonary capillaries have diameters <20–25μm, with sizes down to 3μm in rats (Townsley, 2012), and the ultrasonic insonation method we used detects bubbles with diameters >35μm. Capillaries could thus be blocked by emboli that are smaller than the detection threshold in our experiment. A more sensitive contrast ultrasound method that detect bubbles <10μm in diameter (Le et al., 2021) is needed to further elucidate this point. It is also possible that “frame based” bubble counting would uncover differences in bubble loads that was not picked up by our scoring system (Germonpré et al., 2014).

Additionally, a tendency towards an increase in TPR took place after decompression, indicating the presence of an autonomic compensatory response to the abrupt fall in CO. One may speculate whether there was a reduction of blood volume due to vascular escape, pooling of blood in capillaries, or hypercapnia-induced vascular smooth muscle relaxation during compression and hypothermia (Brickner et al., 1956; Suzuki and Penn, 1965; Tveita et al., 1996). These are changes which cannot be immediately reversed during rewarming (Kondratiev et al., 2006).

There was no indication of cold-induced vasoconstriction during the bottom phase and decompression in our animals, since MAP and TPR were similar in cold rats and controls. It thus appears that the perfusion was unaltered, which is in contrast to a previous report in which wake mildly hypothermic rats (33°C) had reduced nitrogen elimination and washout rate constant (Mack and Lin, 1986). However, the animals in our study were anesthetized, and the effects of anesthesia on vascular tone may explain this apparent discrepancy (Akata and Warltier, 2007).

When the results from this study are interpreted, it should be noted that laboratory animals are not perfect models for human responses (Shanks et al., 2009). While the cardiovascular system of humans and rats are largely similar structurally and functionally (Buetow and Laflamme, 2018), the rodents’ relatively larger body surface area and higher metabolic rate cause them to respond differently from humans to changes in ambient temperature (Maloney et al., 2014). Also, dry diving affects cardiovascular function to a lesser degree than water immersion does (Gaustad et al., 2010), and diving-naïve rats, as the ones in the present study, display different vascular responses than rats that have been exposed to diving regularly (Berenji Ardestani et al., 2019, 2020). Finally, epidemiological data from human divers as well as pre-clinical models show that DCS development is dictated by more factors than bubbles (Cialoni et al., 2017; Lautridou et al., 2017). Additionally, as CO and SV were still falling at the time our experiment was terminated, we do not know how large the final decrease might be had the experiment been extended. Considering these limitations, additional studies that include water immersion, and a longer post-dive observation period, as well as a more severe temperature reduction (“exceptionally cold dives”) are required to identify causal links between cold decompression and DCS.

In conclusion, while a 2°C core temperature reduction during decompression caused CO and SV to fall after dry air diving, no changes were observed in venous bubble grades. However, while we cannot conclude from our data that the DCS risk increased, the post-dive fall in CO and SV could be explained by a fall in LV pre-load secondary to embolic occlusion of pulmonary capillaries. More sensitive bubble detection methods are needed to determine whether there is increased production of smaller gas bubbles (< 35μm) when the body is rewarmed after cold decompression.

## Data Availability Statement

The raw data supporting the conclusions of this article will be made available by the authors, without undue reservation.

## Ethics Statement

The animal study was reviewed and approved by the Norwegian Council for Animal Research.

## Author Contributions

SG and TT designed the study. SG and TK did the experimental work. IE contributed to the manuscript. All authors contributed to the article and approved the submitted version.

## Funding

The study was supported by the Norwegian Petroleum Directorate, Statoil (now Equinor), ExxonMobil, and Gassco under the Competence Program Diving (NUI No. 4600002328) and the Research Council of Norway’s Large-scale Programme for Petroleum Research (Petromaks2 No. 280425). The funders were not involved in the study design, collection, analysis, interpretation of data, writing, or the decision to submit this article for publication.

## Conflict of Interest

SG was employed by the company Møreforsking AS.

The remaining authors declare that the research was conducted in the absence of any commercial or financial relationships that could be construed as a potential conflict of interest.

## Publisher’s Note

All claims expressed in this article are solely those of the authors and do not necessarily represent those of their affiliated organizations, or those of the publisher, the editors and the reviewers. Any product that may be evaluated in this article, or claim that may be made by its manufacturer, is not guaranteed or endorsed by the publisher.
